# Neuroinvasion by *Mycoplasma pneumoniae* in Acute Disseminated Encephalomyelitis

**DOI:** 10.3201/eid1404.061366

**Published:** 2008-04

**Authors:** Bernhard Stamm, Michael Moschopulos, Hansjoerg Hungerbuehler, Jeannette Guarner, Gillian L. Genrich, Sherif R. Zaki

**Affiliations:** * Kantonsspital Aarau, Aarau, Switzerland; † Centers for Disease Control and Prevention, Atlanta, Georgia, USA

**Keywords:** Mycoplasma pneumoniae encephalomyelitis acute disseminated, leukoencephalitis, acute hemorrhagic, Hurst disease, dispatch

## Abstract

We report the autopsy findings for a 45-year-old man with polyradiculoneuropathy and fatal acute disseminated encephalomyelitis after having *Mycoplasma pneumoniae* pneumonia. *M. pneumoniae* antigens were demonstrated by immunohistochemical analysis of brain tissue, indicating neuroinvasion as an additional pathogenetic mechanism in central neurologic complications of *M. pneumoniae* infection.

A 45-year-old, previously healthy man had fever, pain in the extremities, nasal discharge, and cough with nonpurulent sputum. He sought clinical care 1 week after onset of illness because of his deteriorating general state, including a headache and paresthesias in both hands. Bilateral basal pneumonia was diagnosed and treated with clarithromycin. During the ensuing 4 days, a rapidly ascending polyradiculoneuropathy resulted in tetraparesis, followed by facial palsy, ophthalmoplegia, and then paralysis of all cranial nerves. The initially fully alert patient became comatose, and assisted respiration was necessary.

On day 9 of the patient’s illness, an ELISA (Genzyme Diagnostics Virotech, Rüsselsheim, Germany) was performed on serum samples and showed a *Mycoplasma pneumoniae* immunoglobulin (Ig) G antibody titer of 28.2 Virotech-units/mL (VE) (cut-off 9.0–11.0) and an IgM antibody titer of 20.9 VE (cut-off 9.0–11.0). A PCR for *M. pneumoniae* was positive in tracheobronchial secretions on day 12, and complement fixation test (antigen purchased from Virion CH-8803 Rüschlikon, Zürich, Switzerland) showed *M. pneumoniae* antibody titers of 1,280 (serum) and 4 (cerebrospinal fluid) on day 16.

Serologic tests for cytomegalovirus, Epstein-Barr virus, HIV, measles virus, mumps virus, spring-summer encephalitis virus, *Borrelia burgdorferi*, *Brucella* spp., *Legionella* spp., *Treponema pallidum*, and *Toxoplasma gondii* were negative. No herpes simplex virus 1 or 2 was detected by PCR in cerebrospinal fluid, and PCR results were also negative for *Chlamydia pneumoniae* in tracheobronchial secretions.

On day 8, cerebrospinal fluid examinations showed a total cell count of 43/mm^3^ (89% granulocytes and 11% mononuclear cells), total protein 1.3 g/L, and glucose 4.3 mmol/L. On day 15, when the patient was comatose, a total cell count of 794/mm^3^ (84% granulocytes and 16% mononuclear cells), total protein 4.6 g/L, and glucose 1.5 mmol/L. Blood values showed leucocytosis with neutrophilia and mild thrombocytosis of 480 g/L.

A computed tomographic scan on day 15 showed brain edema and multiple inflammatory/demyelination lesions in the subcortical white matter of both hemispheres and within the brain thalami, capsulae internae, midbrain, and pons. Electroneurographic and myographic results showed a severe form of a peripheral axonal neuropathy. No anti-gangliosid (GM) 1 or anti-GM2 antibodies were found in the patient’s serum on day 12. We did not look for GQ1b antibodies.

The clinical diagnosis was polyradiculoneuropathy (atypical Guillain-Barré syndrome) and acute encephalitis as complications of bilateral pneumonia caused by *M. pneumoniae*. In addition to clarithromycin, the patient received amoxicillin and ceftriaxone and was given Ig (0.4 g/kg bodyweight/day for 5 days). He died of intractable cerebral edema on day 17 of illness, 10 days after the onset of neurologic symptoms.

At autopsy, the brain was edematous, weighing 1,560 g. The cerebral meninges were macroscopically unconspicuous. On sectioning, multiple hemorrhagic foci with diameters from 0.5 mm to 2 mm were seen within the white matter of the cerebral and cerebellar hemispheres, the brainstem, and also sparsely within the cortex and basal ganglia. There were mild bilateral basal bronchopneumonia and mild hepatic steatosis.

Microscopically, most of the hemorrhagic foci in the brain consisted of fibrinoid necrosis of the wall of small veins, surrounded by hemorrhagic parenchymal necrosis and a dense annular infiltrate of neutrophils and macrophages (Figure). In less disease-advanced areas, zones of acute perivascular periaxial demyelination were seen around intact vessels; within these same areas, a few vessels were associated with a sleevelike infiltrate of T lymphocytes and macrophages with no evidence of demyelination or necrosis, which probably represented an earlier stage of the process.

**Figure:**
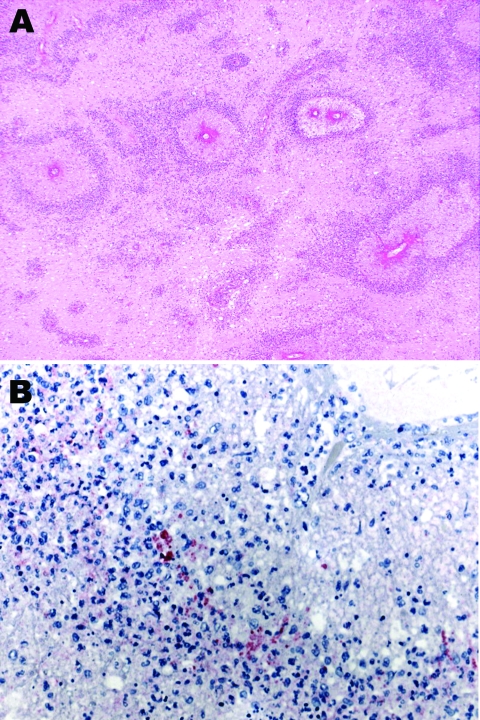
A) Subcortical cerebral white matter with numerous perivascular foci of demyelination and necrosis (hematoxylin and eosin stain, original magnification ×40). B) Immunohistochemical evidence of *Mycoplasma pneumoniae* antigen inside macrophages present in the perivascular inflammatory infiltrate (immunohistochemical assay performed by using the monoclonal anti–*M. pneumoniae* antibody and naphthol fast red as counterstain, original magnification ×100).

Multiple levels of the spinal cord had undergone near total necrosis, extending focally into the spinal nerve roots. Perivascular lesions, as observed in the brain, were also present, albeit in smaller numbers. The spinal meninges were focally occupied by a granulocytic infiltrate. Many meningeal vessels were occluded by fresh thrombi. Tissue samples of the anterior and posterior spinal nerve roots, spinal ganglia, sciatic nerve, and brachial plexus showed acute widespread demyelination, with a dense infiltrate of CD68-positive macrophages and a mild perivascular infiltrate of T lymphocytes and plasma cells.

Immunohistochemical assays were performed by using a polyclonal antibody (Centers for Disease Control and Prevention, Atlanta, GA, USA), an IgG1 kappa monoclonal anti-*M. pneumoniae* antibody (U.S. Biological, Swampscott, MA, USA; cat. no. M9750–12; dilution 1:25), and an immunoalkaline phosphatase with naphthol fast red chromogenic substrate. *M. pneumoniae* antigens were observed inside macrophages present in the inflammatory infiltrate surrounding necrotic vessels within the cerebral hemispheres (including a biopsy specimen obtained 1 day before death), medulla oblongata, and spinal cord (Figure). No *M. pneumoniae* antigens were found in tissue samples of sciatic nerve and lung, probably because of antigen clearance with time, higher drug levels outside the central nervous system (CNS) as an effect of the blood brain barrier, or both. Anatomic diagnoses were acute disseminated encephalomyelitis; acute, inflammatory, demyelinating polyradiculoneuropathy; necrosis of the spinal cord; brain edema; and disseminated intravascular coagulation.

Neurologic disease is a well-known extrapulmonary complication of *M. pneumoniae* infection (*1*,*2*), manifesting as meningoencephalitis, cerebellar ataxia (*3*), brainstem disease (*4*), transverse myelitis (*5*), polyradiculitis (*4*,*6*), or cranial nerve palsies (*4*,*6*). Our findings and previous anatomopathologic reports (*4*,*6*–*8*) support the generally held view that central neurologic complications are the result of disseminated acute encephalomyelitis (ADEM) or of Hurst syndrome, regarded as a hyperacute variant of ADEM. Congestion and edema of the brain, widespread perivenous demyelination mainly in the cerebral and cerebellar white matter, basal ganglia, brainstem, and spinal cord, and in severe cases, necrosis of vessels and perivascular tissue are the histologic characteristics. Rarely, the demyelinating process extends into spinal nerve roots and peripheral nerves.

The pathogenesis of this syndrome is not fully elucidated. An infection-induced, autoimmune hypersensitivity reaction to myelin proteins is the most frequently advanced hypothesis, suggested by the morphologic similarity to experimental allergic encephalitis and the interval between onset of infection and beginning of neurologic symptoms. A T-cell–mediated, delayed type, hypersensitivity reaction, possibly accompanied by an immune-complex type vasculitis (*4*), seems most probable. The occasional demonstration of circulating antibodies against neural tissue supports this hypothesis. Necrosis of the spinal cord found at autopsy in our patient was probably due to secondary ischemia caused by edema and vascular thrombosis, and a similar process may be responsible for the permanent paraplegia of other reported patients with transverse myelitis after *M. pneumoniae* infection (*5*,*6*).

In recent years, the additional or alternative role of invasion of the CNS by the organism itself has gained renewed interest. *M. pneumoniae* RNA can be detected in brain tissue by nucleic acid hybridization (*8*), and the presence of the organism was demonstrated in cerebrospinal fluid by PCR (*1*,*9*–*12*) and by culture (*3*,*12*). As shown in our case, the microbial antigens can be immunohistochemically detected in histopathologically involved areas of the CNS, both in brain biopsy specimens and at autopsy.

Narita et al. searched for *M. pneumoniae* by PCR in the cerebrospinal fluid of 32 patients; all were <15 years and had meningitis, encephalitis, or meningoencephalitis after *M. pneumoniae* infection. Positive results were found in 16 of 32 patients. Of the 16 positive results, 15 were seen in samples from 23 patients who had neurologic complications within 7 days after onset of infection, whereas the search was positive in only 1 of the 9 remaining patients with late onset of neurologic complications (*11*,*13*). Bitnun et al. draw a similar conclusion in their review of childhood encephalitis and *M. pneumoniae*, stating that “direct invasion of the CNS is the probable pathogenetic mechanism in children with a brief (<5 days) duration of the prodromal illness” (*9*). In our patient, neurologic symptoms started 7 days after onset of illness.

The organism is likely present, at least in some patients, in the CNS of those who have an early onset of neurologic complications. To assess its pathogenetic role is difficult. The organism may either cause direct damage or trigger a more violent immunologic reaction. Treatment should in that case aim at arriving rapidly at a sufficient concentration of effective antimicrobial agents within the CNS. The fact that neuroinvasion is more prevalent in patients who have an early onset neurologic complications may also relate to necrosis of vessels, a feature of rapidly progressing disease, which may be caused by or facilitate microbial invasion. Prevention, diagnosis, and treatment of neurologic complications in mycoplasmal infections still pose many problems. Immunohistochemistry may contribute to a better understanding of the pathogenesis of the disease and provide insights on clinical management of patients.
